# Supplementary material for: Transgenic Drosophila lines for LexA-dependent gene and growth regulation
Source: G3 (Bethesda). 2022 Jan 19;12(3):jkac018. doi: 10.1093/g3journal/jkac018 (PMC8895989; doi:10.1093/g3journal/jkac018)
Supplement: jkac018_Supplementary_Table_S1 [file jkac018_supplementary_table_s1.pdf]

Supplementary Table S1. The list of genes and their LexAop-based shRNA transgenic lines generated in this study

Gene-specific shRNAs were amplified from UAS-based shRNA transgenic lines (Bloomington Drosophila Stock Center #) and the forward stem sequences of shRNAs were used to confirm the LexAop-based shRNA plasmids and transgenic lines.

Internal ID	Symbol	Gene	Bloomington ID	shRNA Alleles	Forward Stem Sequence of shRNA	WALEXA shRNA location	Comments
SK001	mCherry	mCherry	35785		CGAGTTCATCTACAAGGTGAA	attP40	
SK002	w	white	33613	HMS00004	CAGCGTCGTCCAGGTGCTGTA	attP40, attP2	one base mismatch in the original UAS stock
IS001	InR	InR	51518	HMS03166	CCGGACGACCGTCAAAGGTTA	attP40, attP2	
IS002	chico	chico	36665	HMS01553	TTGGACAAACTACTAGTTCTA	attP40	
IS003	Akt1	Akt1	33615	HMS00007	ATCGGACTTCCTGCTCAACAA	attP40, attP2	
IS004	Pten	Phosphatase and tensin homolog	33643	HMS00044	TAGGCATGGTAGAATGTTCAA	attP40	
IS005	Tsc1	Tsc1	52931	HMC03672	TTGGCTGTACAAGATCGAGAA	attP40	
IS006	gig	gigas	34737	HMS01217	CAAGTTCAAGTTGTTCCTCAA	attP40	
IS007	Rheb	Ras homolog enriched in brain	33966	HMS00923	CAGGTTTAAGTTAGTACGTTT	attP40	
IS009	S6k	Ribosomal protein S6 kinase	57016	HMS04459	CTGGACGAGATGCTAACAAAT	attP40	
IS010	AMPKalpha	AMP-activated protein kinase α subunit	57785	HMC04979	CCCGAGTACCTCAACAAACAA	attP40	
PP001	Pp1-87B	Protein phosphatase 1 at 87B	32414	HMS00409	CGGATTCTACGACGAATGCAA	attP40	
PP002	CG12091	CG12091	40936	HMS02184	AACCGACGGAGTGTTCGACAA	attP40	
PP003	fig	fos intronic gene	65056	HMC06012	CACCGATGGCTTGTTCGATAA	attP40	
PP004	Pp2C1	Protein phosphatase 2C	40827	HMS01887	AACGTTATTGCTGTAAGCAAA	attP40	
PP005	flw	flapwing	38336	HMS01803	AAGTACAGCGATGTTAAGCAA	attP40	
PP006	Pgam5	Phosphoglycerate mutase 5	33346	HMS00212	CAGGCGGAGTTTACTGGGAAA	attP40	
PP007	mts	microtubule star	38337	HMS01804	AAGCAACAAGAAGAACTACAA	attP40	
PP008	CG9236	Calcium and integrin binding family member 2	38532	HMS01745	CAGCAGATTGCCGACAAGGTA	attP40	
PP009	CG3632	CG3632	38341	HMS01808	AAGGGACGAGCAGCTACTCAA	attP40	noisy sequencing result
PP011	Ppm1	Ppm1	41987	HMS02386	CAGACGCTGTTGGCACTACAA	attP40	
PP012	Pp4-19C	Protein phosphatase 19C	38372	HMS01841	CCGGGTGAAGATGTCCGACTA	attP40	noisy sequencing result
PP013	Pdp	Pyruvate dehydrogenase phosphatase	38972	HMS01888	TTCCTCAAGTATGTCAATCAA	attP40	
PP014	CG17746	CG17746	33347	HMS00214	CAGGCGACTAACAATGATCAA	attP40	
PP015	CG7115	CG7115	39065	HMS01985	TACGATAAGAGTCCCTATTTA	attP40	
PP016	CG8584	CG8584	57005	HMC04448	CCGGACTATGTGCTCAATATT	attP40	
PP017	Pp1-Y1	Protein phosphatase 1, Y-linked 1	58098	HMJ22013	CAGGAACGGACTTCTATGCGA	attP40	
PP018	PpD6	Protein phosphatase D6	62849	HMC05322	TCGAGGTGTTAGCTATCTCTA	attP40	
PP019	Phlpp	PH domain leucine-rich repeat protein phosphatase	57399	HMC04703	AAGGACGACGAGTTCGTACTA	attP40	
PP020	PpV	Protein phosphatase V	57765	HMC04959	CAAGTGGATAGAAGACGTGAA	attP40	
PP021	PpD3	Protein phosphatase D3	57307	HMS04508	ACGGTCAATTCTACGACTTGA	attP40	
PP022	CanA-14F	Calcineurin A at 14F	38966	HMS01880	CAGTTGGTAGTCGACAGTTAA	attP40	
PP024	Pten	Phosphatase and tensin homolog	33643	HMS00044	TAGGCATGGTAGAATGTTCAA	attP40	
PP025	Fcp1	TFIIF-interacting CTD phosphatase	32925	HMS00716	TAGCTGTTAATATGTACTTTA	attP40	
PP026	Pp1-Y2	Protein phosphatase 1, Y-linked 2	57236	HMC04622	AAGCGACAACTAATTACTATA	attP40	
PP028	Pp2B-14D	Protein phosphatase 2B at 14D	40872	HMS02039	TAGCTTAAGTTGTAATGTATA	attP40	
PP030	tws	twins	36689	HMS01578	CCGGAAGTTGTCTCAGTCAAA	attP40	
PP031	Ssu72	Ssu72 CTD phosphatase	38344	HMS01811	CAGAGTATTAGAGTAAGACAA	attP40	
PP032	Pp1-13C	Protein phosphatase 1 at 13C	32465	HMS00465	AAGCGCGAATGTAGAGTATTA	attP40	
PP033	PpN58A	Protein phosphatase N at 58A	57402	HMC04706	CCGACCAAGTTCTACCTACTA	attP40	
PP034	CG6036	CG6036	65115	HMC06011	CACGATGAATTCATAGTTGTA	attP40	
PP036	Sur-8	Sur-8	41883	HMS02299	TTGTATGTTAGTCTAAGCTAA	attP40	one base mismatch in the original UAS stock
PP038	Pp1alpha-96A	Protein phosphatase 1α at 96A	40906	HMS02154	CCGGGAAAGAATGTTCAGCTA	attP40	
PP040	ttm2	tiny tim 2	61973	HMJ23599	ACGCTACTCGACGTACGAGAA	attP40	
PP041	CG5830	CG5830	34079	HMS01077	AAGCAAGTAAATTATACATAA	attP40	
PP042	CG10376	CG10376	41907	HMS02300	TACGTTATTGTTGTAATGTAA	attP40	
PP043	CG10417	CG10417	39051	HMS01971	ATCGATAGTCAGCATACTAAA	attP40	
PP045	PpD5	Protein phosphatase D5	77379	HMC06510	ACGAGTGCAAGCGACGATATA	attP40	
