# Supplementary material for: Transgenic Drosophila lines for LexA-dependent gene and growth regulation
Source: G3 (Bethesda). 2022 Jan 19;12(3):jkac018. doi: 10.1093/g3journal/jkac018 (PMC8895989; doi:10.1093/g3journal/jkac018)
Supplement: jkac018_Supplementary_Figure_S1 [file jkac018_supplementary_figure_s1.pdf]

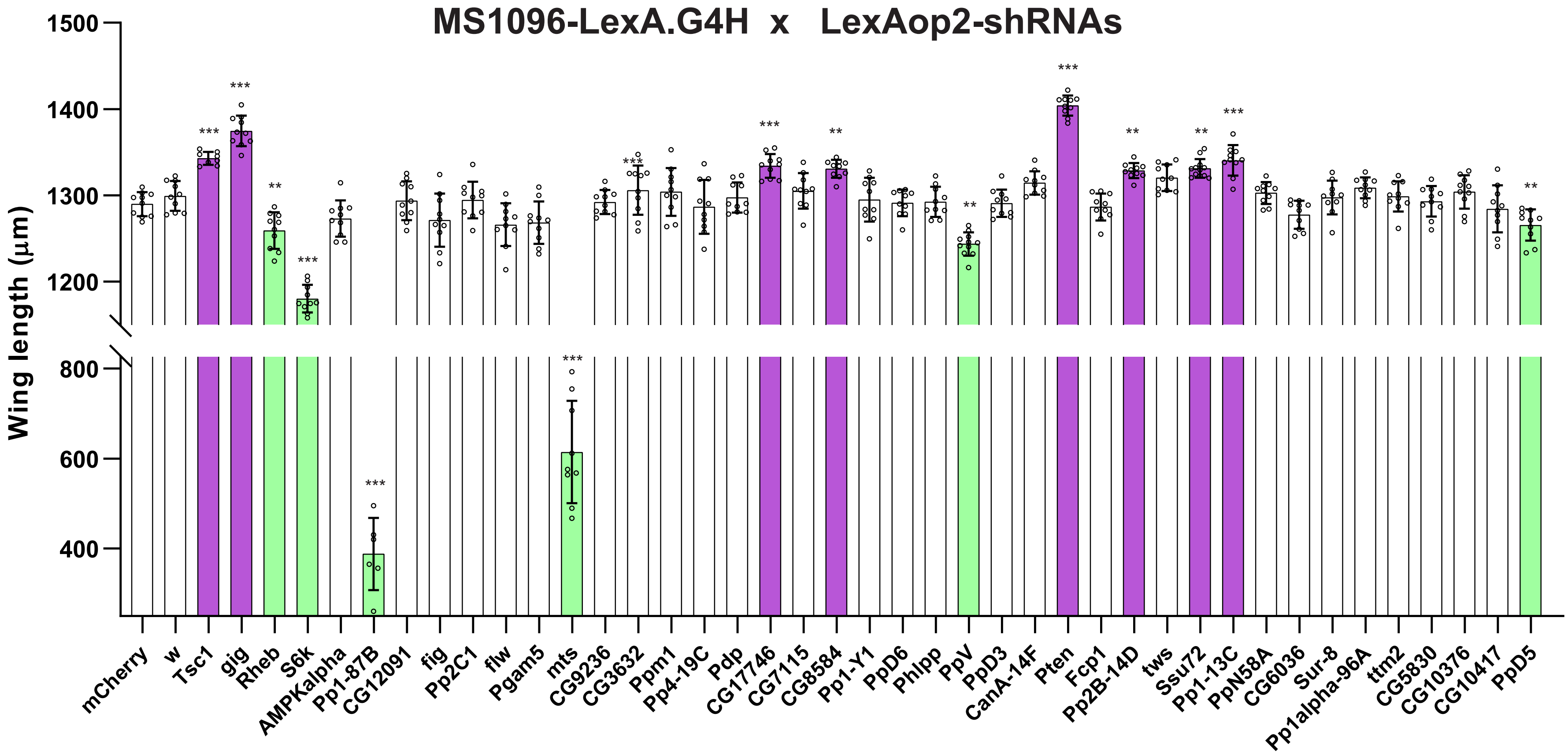

Supplementary Figure S1. A genetic screen of growth regulators for Drosophila wing development by LexA/LexAop system. Quantification of wing length in flies expressing shRNAs for protein phosphatases and additional insulin signaling component genes. The purple bars indicate that gene-specific shRNA expression increased wing length compared to the control *mCherry* shRNA while the green bars designate decreased wing length (P<0.01, student's t-test). The error bars are standard deviations. The average lengths are based on n = 8 wings in each genotype of female progeny from the indicated crosses above. ** denotes P<0.01, and *** indicates P<0.001.
